# Multilocus DNA Barcoding Resolves Area-Based Genetic Variation in Freshwater Shrimps (*Macrobrachium* sp. and *Caridina* sp.) from Saline and Non-Saline Habitats in Northeastern Thailand

**DOI:** 10.3390/biology15171559

**Published:** 2026-09-06

**Authors:** Juthaporn Saengprajak, Noppakun Pakdeenarong

**Affiliations:** Department of Biology, Faculty of Science, Mahasarakham University, Kham Riang, Kantharawichai District, Maha Sarakham 44150, Thailand; juthaporn.s@msu.ac.th

**Keywords:** DNA barcoding, species delimitation, multilocus phylogeny, *COI*, *16S rRNA*, *18S rRNA*, ITS, *Macrobrachium*, *Caridina*

## Abstract

Freshwater prawns are important components of freshwater ecosystems, yet their diversity and genetic structure in northeastern Thailand remain insufficiently documented, particularly across habitats differing in soil salinity. This study assessed freshwater prawn diversity and phylogenetic relationships in saline and non-saline habitats using an integrative taxonomic approach combining morphological examination with mitochondrial and nuclear DNA barcoding. A total of 225 mature specimens were collected from 15 sampling sites in Maha Sarakham province (saline habitats) and Mukdahan province (non-saline habitats). Representative specimens were examined morphologically and characterized using four barcode loci: cytochrome c oxidase subunit I (*COI*), 16S ribosomal RNA (*16S rRNA*), 18S ribosomal RNA (*18S rRNA*), and the internal transcribed spacer (ITS). Morphological assessment identified three taxa, *Macrobrachium lanchesteri*, *Macrobrachium niphanae* and *Caridina* sp. Molecular analyses recovered three principal lineages broadly corresponding to these taxonomic groups, although the resolution varied among loci. *COI* provided consistent species-level discrimination, while *16S rRNA* revealed additional genetic variation within *Macrobrachium*. The conserved *18S rRNA* marker supported separation of the major taxonomic lineages, whereas ITS contributed complementary sequence variation for lineage assessment. The concatenated multilocus dataset provided the most stable phylogenetic reconstruction, with strong support for the principal lineages. Samples from saline and non-saline habitats were not consistently separated, suggesting that salinity was not the primary factor underlying the observed genetic structure. Overall, integrating morphological and multilocus molecular evidence provided a robust framework for assessing freshwater prawn diversity and phylogenetic relationships and establishes a baseline for future taxonomic, phylogeographic, and conservation studies in the Mekong region.

## Simple Summary

Freshwater prawns are an important part of rivers and streams because they help maintain healthy aquatic ecosystems and provide food and income for local communities. However, many freshwater prawn species are difficult to identify because different species often look very similar. This study explored freshwater prawn diversity in northeastern Thailand by comparing areas with naturally saline soil and areas with normal freshwater conditions. We collected prawns from 15 locations and identified them using both their physical characteristics and their genetic information. Three known species were identified. We also found that areas without saline soil supported a greater variety of freshwater prawns than saline areas. In addition, we compared four different genetic methods and found that combining more than one method improved the accuracy of species identification. These findings provide new information about freshwater prawn diversity in Thailand and highlight the importance of combining traditional observations with genetic tools. The results will support future conservation programs, improve biodiversity assessments, and provide a scientific foundation for describing new species and protecting freshwater ecosystems in the Mekong region.

## 1. Introduction

Freshwater ecosystems in Southeast Asia represent some of the most biodiverse aquatic environments on Earth, harboring a remarkable richness of decapod crustaceans that are integral to both ecological function and human livelihoods [1,2]. Among these, freshwater shrimps belonging to the families Palaemonidae and Atyidae constitute dominant invertebrate groups in streams, rivers, ponds, and paddy fields across the region [3]. The genus *Macrobrachium* Spence Bate, 1868 (Palaemonidae), with over 271 species described globally, is one of the most speciose freshwater decapod genera, exhibiting its highest diversity across the river basins of the Oriental Region [4]. In Thailand alone, 34 species have been reported to date, with the Chao Phraya and Mekong River Basins serving as principal hotspots of species richness and endemism [5,6]. The genus *Caridina* H. Milne Edwards, 1837 (Atyidae) is equally diverse, comprising more than 300 valid species worldwide and at least 15 species recorded from Thailand, with recent discoveries of land-locked endemic taxa from the Middle Mekong Basin further expanding its known diversity [7,8]. Collectively, these shrimps provide critical ecosystem services including nutrient cycling and serving as prey for higher trophic levels, while also supporting significant commercial fisheries, aquaculture industries, and a growing ornamental trade across the region [9].

Salinity is an important ecological factor shaping the distribution, dispersal capacity, and evolutionary differentiation of freshwater shrimps. In palaemonid prawns, populations occupying estuarine, brackish, and inland freshwater habitats may experience different osmotic regimes that influence larval survival, connectivity, and ultimately genetic structure. Comparative genomic studies of *Macrobrachium* species occupying different salinity niches have further demonstrated species-specific transcriptional responses and signatures of selection in genes involved in osmoregulation, water balance, egg size, and larval development, indicating that adaptation to contrasting osmotic environments can contribute to evolutionary divergence [10].

Among the most ecologically prevalent species in mainland Southeast Asia is *Macrobrachium lanchesteri* (De Man, 1911), a small, translucent palaemonid prawn that dominates diverse freshwater habitats including rivers, lakes, ponds, and paddy fields throughout Thailand, Malaysia, Singapore, and southern China [11]. *Macrobrachium nipphanae* represents another taxon of taxonomic interest within the Thai *Macrobrachium* fauna. Described from central and northeastern Thailand, it belongs to a group of small-bodied freshwater prawns whose morphological similarity to related species, particularly within the *M. sintangense* and *M. lanchesteri* complexes, renders visual identification highly problematic [12]. *Caridina* sp. is a widespread species recorded across Southeast Asian rivers and estuaries, recognized by its slender, elongated rostrum and relatively transparent body [13]. The presence of multiple morphologically similar species within the genus, combined with the discovery of several cryptic taxa in recent years, has demonstrated that current morphological keys are insufficient for confident species-level identification across the full range of *Caridina* diversity in Southeast Asia [14]. The accurate identification of *Macrobrachium* and *Caridina* species using morphological characters alone faces serious and well-documented constraints. Primary among these is the pronounced intraspecific variability exhibited by many taxa, driven by sexual dimorphism, ontogenetic change, and environmental plasticity [15].

The development of DNA barcoding and multilocus molecular approaches has provided powerful solutions to the identification challenges described above. By standardizing the use of short, informative gene regions to discriminate species, molecular barcoding enables rapid, repeatable, and objective species assignments that transcend the limitations of morphological variation [16].

The mitochondrial *COI* gene for discriminating species across both *Macrobrachium* and *Caridina* in India, revealing clear intergeneric and interspecific barcode gaps and providing reference sequences for future identifications [17]. Consequently, Saengprajak et al. [18] analyzed concatenated *COI* with morphological evidence and separated *Macrobrachium* and *Caridina* lineages and that study reported four morphologically identified taxa and recovered several *COI*-associated sequence clusters, including records that were provisionally interpreted as novel cryptic freshwater shrimp species *Macrobrachium* and *Caridina* lineages. ITS2 barcoding on *M. lanchesteri* populations and resolved nine distinct haplotypes with strong partitioning of genetic variation among geographic groups, demonstrating that nuclear markers can complement mitochondrial data to reveal population-level structuring masked by morphology alone. To resolve these issues, an integrative multilocus approach combining mitochondrial and nuclear markers offers superior taxonomic resolution compared with any single-marker strategy. The *16S rRNA* gene offers complementary phylogenetic signal, particularly for resolving deeper nodes. The *18S rRNA* gene, though highly conserved, contributes to higher-level phylogenetic placement and aids in distinguishing genus-level boundaries. The ITS (internal transcribed spacer) region provides fine-scale intraspecific and interspecific variation useful for detecting population structure and hybridization, as demonstrated by its ability to resolve nine *M. lanchesteri* haplotypes undetectable by morphology alone.

This study therefore aims to apply a multilocus DNA barcoding approach targeting COI, 16S rRNA, 18S rRNA, and ITS markers to resolve the area-based genetic variation in *Macrobrachium* and *Caridina* from saline and non-saline habitats in Northeastern Thailand, contributing robust taxonomic foundations for future ecological and conservation research in this ecologically critical region.

## 2. Materials and Methods

**Figure 1:**
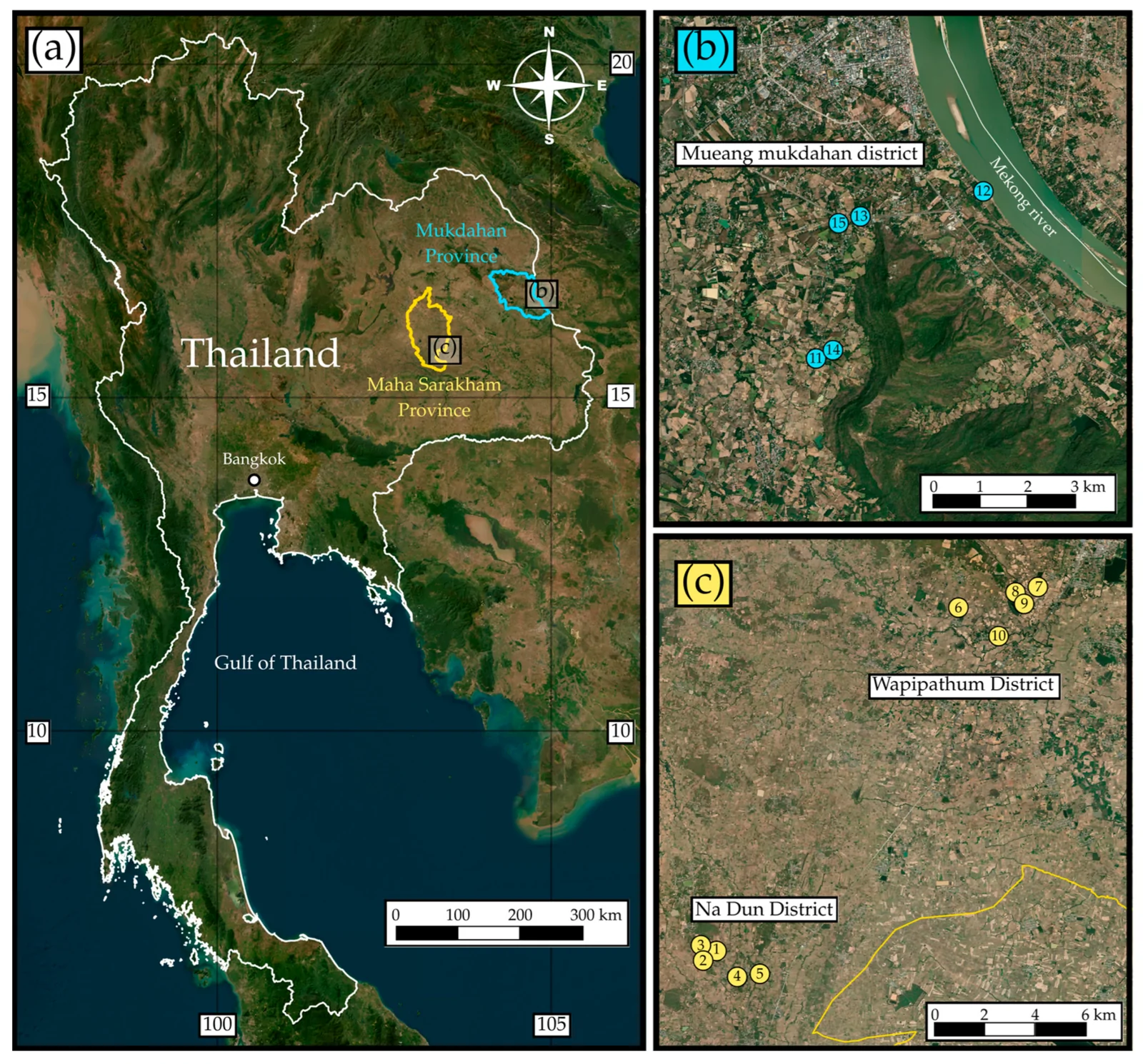
The sampling sites 15 localities across Mahasarakham (yellow area)and Mukdahan (bule area) provinces between March through April 2026. (**a**) The position of 2 province deposit in Thailand. (**b**) Localization of 5 sites in Mukdahan province. (**c**) Localization of 5 sites in Nadun distrct and 5 sites in Wapipathum district. The number were shown in Table 1. The sampling map was generated using open source QGIS software version 4.0.3 (QGIS Development Team, 2025; [19]). https://www.qgis.org, accessed on 7 July 2026.

Shrimp specimens were obtained from 15 localities across Mahasarakham and Mukdahan Provinces from March through April 2026, as described in Figure 1; Table 1 represents the geographic distribution of the sampling sites. The sampling map was generated using open source QGIS software version 4.0.3 (QGIS Development Team, 2025 [19]). https://www.qgis.org, accessed on 7 July 2026.

### 2.1. Ethics Statement

This research was carried out in line with the ethical guidelines set by the Institute of Animals for Scientific Purposes Development, Thailand. The study received approval from the Ethics Committee of the Mahasarakham University Institute Animal Care and Use Committee (license number IACUC-MSU-001-024/2026), which confirmed full adherence to the regulations governing shrimp handling; moreover, the shrimp were euthanized in iced boxes and subsequently stored in 75% and 95% (*v*/*v*) ethanol.

Field sampling was conducted at 15 sites in two months between March through April 2026. Water temperature, pH, and salinity were measured using a water quality meter. Water temperature (°C) was measured with water quality instrument ModelWater temperature (°C) was measured with a digital thermometer; pH (pH units) with a pH meter (Hanna Instruments, HI98127, Smithfield, RI, USA); and salinity (ppt) with a salinity meter (ATAGO PAL-ES2, Tokyo, Japan).

A total of 225 mature shrimps were collected; at each site, five individuals per species were sampled using morphological identification. Live specimens were transferred to Mahasarakham university laboratory. The shrimp barcoding studies was to explain the rationality of 5 replicates per species for baseline genetic diversity screening according to Chakraborty et al. [10]. This sampling density follows common practice in regional DNA barcoding surveys (typically 5–10 individuals per species [20]. Morphological identification was conducted according to standardized procedures according to Cai and Ng 2002 and Cai, 2025 [21,22], emphasizing rostral traits, pereopods, pleopods, and reproductive characteristics.

### 2.2. Morphometric Measurement

Quantitative rostral traits were measured following standardized landmarks commonly used in *Macrobrachium* and *Caridina* taxonomy [23]. Rostrum length (RL) was measured as the straight-line distance from the rostral origin (base) to the tip of the rostrum. Carapace length (CL) was measured as the straight-line distance from the posterior margin of the orbit to the posterior mid-dorsal margin of the carapace. All specimens were photographed under a stereo microscope (ZEISS Axiostar Plus C) together with a calibrated scale bar included in each image field. Linear measurements were obtained from the digital images using pixel-based image analysis calibrated against the embedded scale bar for each photograph, with each measurement taken in triplicate and averaged to minimize measurement error. The dentition formula was recorded as (number of dorsal teeth)/(number of ventral teeth), counted along the entire rostral margin from the base to the tip under magnification sufficient to resolve individual teeth. From the measured RL and CL values, the rostrum-to-carapace length ratio (RL/CL) was calculated for each specimen as an additional quantitative index of rostral shape. Then specimens were stored long-term in 75% and for molecular analysis in 95% (*v*/*v*) ethanol, respectively.

### 2.3. Statistical Analysis

Quantitative rostral metrics (CL, RL, and RL/CL ratio) were compared among the three sampling areas (Na Dun and Wapipathum districts, Maha Sarakham Province and Mukdahan Province separately for each species using one-way ANOVA, followed by Tukey’s HSD post hoc test for pairwise comparisons (*p* < 0.05).

### 2.4. DNA Extraction and DNA Quality Assessment

Genomic DNA was extracted using the Genomic DNA Isolation Kit (BIO-HELIX Co., Ltd., New Taipei City, Taiwan) according to the manufacturer’s instructions. Approximately 0.3–0.5 g of shrimp tissue was excised from the whole body of each specimen and transferred into sterile 1.5 mL microcentrifuge tubes for DNA extraction.

The quality, concentration, and purity of the extracted genomic DNA were evaluated following standard procedures for nucleic acid quality assessment [24]. DNA concentration and purity were determined using a NanoDrop spectrophotometer (NND-1 NDL-PLUS-GL, Thermo Fisher Scientific Co., Ltd., Waltham, MA, USA) by measuring absorbance at 230, 260, and 280 nm. DNA purity was assessed using the A260/A280 and A260/A230 absorbance ratios. An A260/A280 ratio of approximately 1.8 was considered indicative of high-quality DNA, whereas values below 1.6 suggested contamination by proteins or phenolic compounds, and values above 2.0 indicated possible RNA contamination. DNA samples were diluted with nuclease-free water (Invitrogen™, Thermo Fisher Scientific Co., Ltd., Waltham, MA, USA) to a working concentration of 50 ng μL^−1^ and stored at −20 °C until further analysis.

The integrity of genomic DNA was verified by electrophoresis on a 1.0% (*w*/*v*) agarose gel prepared in 1× Tris–Borate–EDTA (TBE) buffer, following the protocol of [25]. Briefly, 5 μL of each DNA sample was mixed with 3 μL of Novel Juice non-toxic DNA loading dye and separated by electrophoresis at 120 V for 35 min. DNA fragments were visualized under UV illumination using a GelDoc Go Gel Imaging System (Bio-Rad, Hercules, CA, USA) after staining with ViSafe Green Gel Stain (Vivantis^®^, Subang Jaya, Malaysia). A 100 bp DNA ladder Ready-to-Use (RTU) (GeneDirex, Inc., Taoyuan, Taiwan) was included as a molecular size marker to confirm successful DNA extraction and assess genomic DNA integrity.

### 2.5. PCR Amplification of DNA Barcode Loci

The *COI*, 16S rRNA, 18S rRNA, and ITS DNA barcode regions were amplified by polymerase chain reaction (PCR) using the primer pairs listed in Table 2. PCR amplifications were performed using a Veriti™ Thermal Cycler (Applied Biosystems, Singapore). The primers were synthesized by U2Bio Co., Ltd. (Bangkok, Thailand), and the primer sequences were selected from previously published DNA barcoding protocols [26]. Each PCR was performed in a final volume of 25 μL containing 15 μL of 1.2× OmniPCR Supermix (BioHelix, Beverly, MA, USA), 1.5 μL of forward primer (50 pmol μL^−1^), 1.5 μL of reverse primer (50 pmol μL^−1^), 1 μL of genomic DNA (50–100 ng), and nuclease-free water to the final volume. The same reaction mixture was used for all four DNA barcode loci. The PCR cycling program consisted of an initial denaturation at 95 °C for 5 min, followed by 35 cycles of denaturation at 95 °C for 30 s, primer annealing for 30 s at a locus-specific temperature, and extension at 72 °C for 1 min. The annealing temperatures were 52 °C for COI, 53 °C for 16S rRNA, and 55 °C for both 18S rRNA and ITS (Table 2), followed by a final extension at 72 °C for 10 min.

PCR conditions were optimized based on published DNA barcoding protocols and further validated through preliminary amplification assays to ensure specific and reproducible amplification. Negative controls containing all PCR components except template DNA were included in each PCR run, and all amplifications were performed in duplicate. PCR products were verified by electrophoresis on 1% agarose gels, and successfully amplified products were purified and commercially sequenced by U2Bio Co., Ltd. (Bangkok, Thailand) using the corresponding PCR primers.

### 2.6. Data Analysis

Phylogenetic relationships among 36 sequences from 9 specimens, generated in this study were investigated using DNA barcode sequences. The *COI*, *16S rRNA*, *18S rRNA*, and ITS sequences generated in this study were compared with sequences available in the NCBI GenBank database using the BLASTn algorithm (https://blast.ncbi.nlm.nih.gov/Blast.cgi, accessed on 4 June 2026) [30]. BLASTn searches were used to evaluate sequence similarity and to identify the closest available nucleotide sequences based on percentage identity, query coverage, aligned sequence length, and E-value. The closest matches and their corresponding GenBank accession numbers are presented in Table 2. For *COI*, *16S rRNA*, and *18S rRNA*, sequence similarity thresholds were used to support species identification. No fixed BLAST similarity threshold was applied to ITS sequences because of their limited taxonomic resolution in public databases. Instead, ITS was used primarily to assess sequence variation and delineate phylogenetic lineages in combination with the other barcode loci. The resulting sequences were examined for sequence quality, and consensus sequences were generated from the obtained chromatograms before subsequent analyses. For ITS sequences, quality control included manual inspection of chromatograms, trimming of low-quality terminal regions, and assessment of sequence quality following alignment. Poorly aligned or ambiguous regions were excluded before phylogenetic reconstruction. The consensus sequences generated in this study were deposited in the NCBI GenBank database, and the corresponding accession numbers are provided in Table 2.

Multiple sequence alignments were generated using Clustal Omega (https://www.ebi.ac.uk/jdispatcher/msa/clustalo, accessed on 4 June 2026) [31]. Phylogenetic analyses were conducted using molecular evolutionary genetics analysis version 11 (MEGA11) [32]. Phylogenetic relationships were reconstructed using the Maximum Likelihood (ML) method based on the best-fit nucleotide substitution model selected according to the Bayesian Information Criterion (BIC) implemented in MEGA11. The selected nucleotide substitution model was subsequently used for ML tree reconstruction in MEGA11. The robustness of the inferred phylogenetic relationships was assessed using 1000 bootstrap replicates [33]. Bootstrap support values were interpreted according to [34], with values >85% considered strong support, 70–85% moderate support, 50–69% weak support, and <50% poor support.

## 3. Results

### 3.1. Water Quality

The environmental data revealed a clear separation between the two regions at the time of sampling. Maha Sarakham sites associated with saline soils had salinities of 0.100–0.550 ppt (mean 0.268 ppt) and electrical conductivity of 402–2050 µS/cm (mean 791 µS/cm). In contrast, the Mukdahan sites had salinities of 0–0.050 ppt (mean 0.032 ppt) and conductivity of 245–305 µS/cm (mean 276 µS/cm). Thus, mean salinity and conductivity were approximately 8.4- and 2.9-fold higher, respectively, in Maha Sarakham province (Table 3).

### 3.2. Rostral Characteristics

#### 3.2.1. *Caridina* sp.

**Figure 2:**
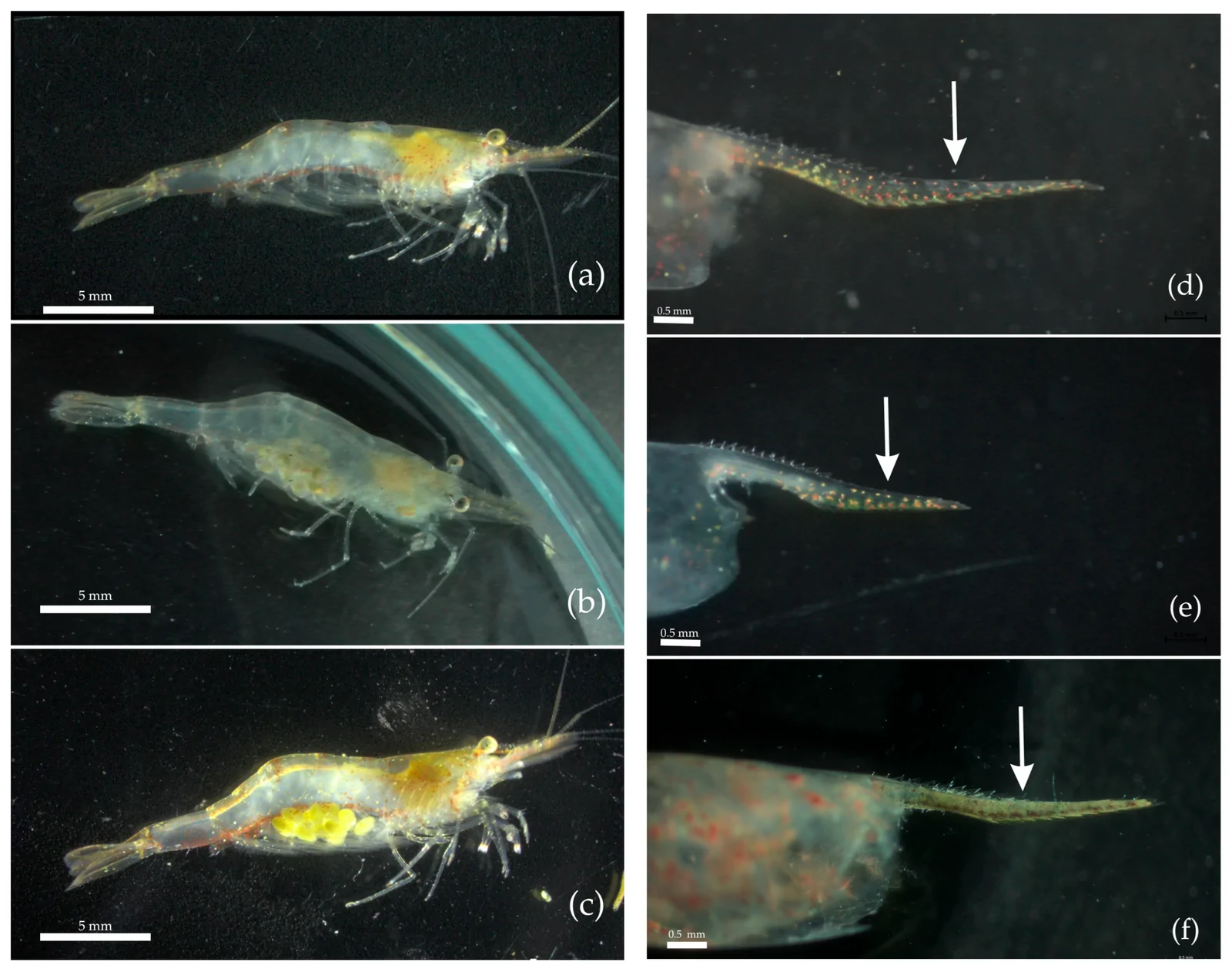
External morphology of representative *Caridina* specimens collected from three sampling sites in northeastern Thailand. (**a**–**c**) Lateral view of whole-body morphology of specimens collected from Na Dun District, Mahasarakham Province (**a**), Wapipathum District, Mahasarakham Province (**b**), and Mukdahan Province (**c**). (**d**–**f**) Enlarged view of the rostrum of the corresponding specimens showing variation in rostral shape (white arrow). Scale bars: 5 mm (**a**–**c**) and 0.5 mm (**d**–**f**).

Shrimps collected from the three sampling sites exhibited generally similar external morphology, including body proportions, eye size, and appendage structure (Figure 2a–c). However, slight variation was observed in body coloration, chromatophore distribution, and rostral morphology. Specimens from Na Dun District, Maha Sarakham Province, possessed a relatively slender rostrum and a conspicuous yellow-orange chromatophore patch on the cephalothorax, accompanied by numerous red chromatophores distributed along the cephalothoracic region (Figure 2a,d). The rostral formula was 13–20/6–12 teeth. Rostrum length was 6–7 mm (6.53 ± 0.25 mm) as shown in Table 4.

In contrast, specimens from Wapipathum District, Maha Sarakham province, exhibited a comparatively shorter rostrum, paler body coloration, fewer visible chromatophores, and a slightly broader cephalothorax (Figure 2b,e). The rostral formula was 13–16/6–12 teeth. Rostrum length was 3–4 mm (3.59 ± 0.80 mm) as shown in Table 4.

Specimens collected from Mukdahan Province possessed the slenderest rostrum among the examined populations and displayed less distinct yellow-orange pigmentation but a greater abundance of red chromatophores distributed over the cephalothorax and rostrum (Figure 2c,f). Despite these observable differences, the external morphological characters largely overlapped among populations and did not provide sufficient diagnostic features for reliable species delimitation. Such phenotypic variation may reflect geographic variation, environmental influences, ontogenetic differences, or intraspecific polymorphism rather than species-level divergence. The rostral formula was 13–16/10–12 teeth. Rostrum length was 5–6 mm (5.76 ± 0.79 mm) as shown in Table 4.

#### 3.2.2. *Macrobrachium lanchesteri* (De Man, 1911)

**Figure 3:**
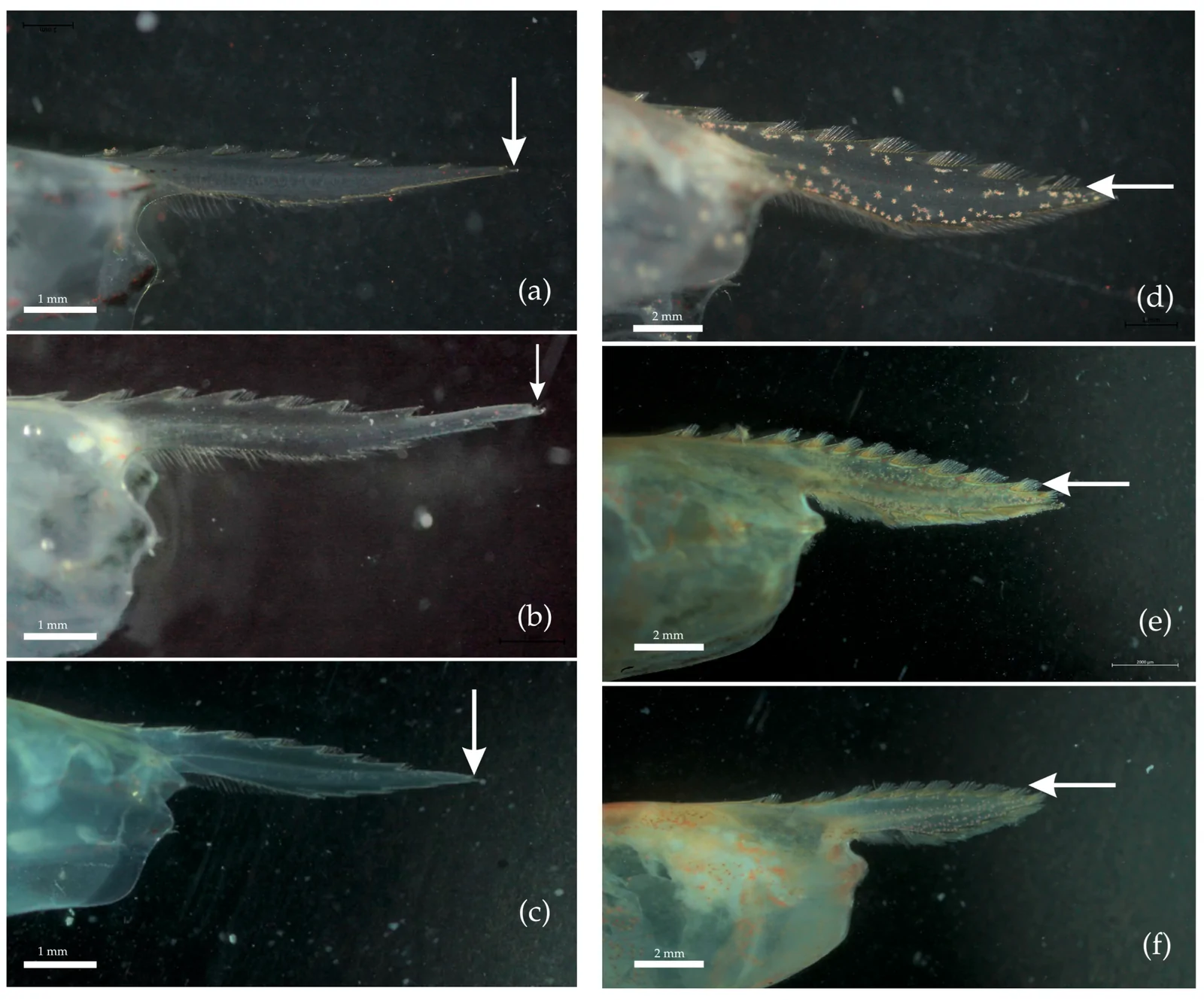
Comparison of rostral morphology among freshwater shrimp populations collected from northeastern Thailand. (**a**) *M. lanchesteri* from Na Dun District, Mahasarakham province showing a relatively long and slender rostrum with numerous dorsal and ventral teeth. The tip of rostrum was different (white arrow) (**b**) *M. lanchesteri* from Wapipathum District, Maha Sarakham province exhibiting a shorter and more robust rostrum with fewer ventral teeth. (**c**) *M. lanchesteri* from Mukdahan province displaying an intermediate rostral morphology with a slender rostrum and moderately developed dorsal dentition. Scale bars = 1 mm. (**d**) Rostrum of *M. niphanae* from Na Dun District, Maha Sarakham province, displaying an elongate, slender rostrum with numerous dorsal and ventral teeth and dense white chromatophores on the dorsal surface. (**e**) Rostrum of *M. niphanae* from Wapipathum District, Maha Sarakham province, displaying a comparatively shorter and broader rostrum with prominent dorsal teeth and relatively sparse chromatophore pigmentation. (**f**) Rostrum of *M. niphanae* from Mukdahan province, displaying an intermediate rostral morphology with evenly distributed dorsal teeth and reddish chromatophores concentrated near the rostral base. The number of dorsal teeth was different (white arrows). Scale bars = 2 mm.

The rostrum exhibited noticeable variation among the three populations despite their generally similar external morphology (Figure 3). Differences were primarily observed in rostral length, curvature, and the arrangement of dorsal and ventral teeth.

*Macrobrachium lanchesteri* from Na Dun District, Mahasarakham Province possessed a relatively long, slender, and slightly upward-curved rostrum, extending well beyond the antennular peduncle (Figure 3a). The dorsal margin bore numerous small teeth distributed along most of the rostral length, whereas the ventral margin contained a continuous row of fine teeth extending nearly to the distal tip. The distal portion gradually tapered to an acute apex. The rostral formula was 7–10/3–4 teeth. Rostrum length was 5–8 mm (6.27 ± 0.67 mm) as shown in Table 4.

In contrast, specimens from Wapipathum District, Mahasarakham Province exhibited a shorter and comparatively stouter rostrum (Figure 3b). The dorsal teeth were larger and more widely spaced than those observed in the Na Dun specimens, while the ventral teeth were fewer in number and confined to the proximal half of the rostrum. The distal region appeared broader with a less pronounced taper. The rostral formula was 7–8/3–4 teeth. Rostrum length was 3–4 mm (3.57 ± 0.08 mm) as shown in Table 4.

*Macrobrachium lanchesteri* from Mukdahan Province possessed an intermediate rostral morphology (Figure 3c). The rostrum was relatively slender but shorter than that of the Na Dun population. The dorsal teeth were moderately developed and evenly distributed along the dorsal margin, whereas the ventral teeth were less conspicuous and restricted to the basal region. The rostral apex was narrow and gently curved upward. The rostral formula was 7–8/3–4 teeth. Rostrum length was 8–9 mm (8.38 ± 0.03 mm) as shown in Table 4.

#### 3.2.3. *Macrobrachium niphanae*

*Macrobrachium niphanae* collected from Na Dun District, Maha Sarakham province exhibited the longest and most slender rostrum among the examined populations (Figure 3d). The dorsal margin bore numerous small, evenly spaced teeth extending almost to the distal tip, while the ventral margin contained a continuous series of fine teeth. Numerous white chromatophores were distributed over the dorsal surface of the rostrum, giving it a distinctly speckled appearance. The rostral formula was 7–13/2–3 teeth. Rostrum length was 10–14 mm (11.84 ± 1.28 mm) as shown in Table 4.

*Macrobrachium niphanae* from Wapipathum District, Maha Sarakham province possessed a comparatively shorter and broader rostrum (Figure 3e). The dorsal teeth were larger, more robust, and slightly more widely spaced than those observed in the Na Dun population. The ventral teeth were less conspicuous and appeared to be restricted to the proximal half of the rostrum. Pigmentation was generally weaker, and the dorsal surface lacked the dense white chromatophores observed in the Na Dun specimen. The rostral formula was 7–12/2–3 teeth. Rostrum length was 7–8 mm (7.07 ± 0.23 mm) as shown in Table 4.

*Macrobrachium niphanae* from Mukdahan province exhibited an intermediate rostral morphology (Figure 3f). The rostrum was moderately slender with a gently tapering apex. Dorsal teeth were regularly arranged but less prominent than those of the Wapipathum population, whereas the ventral teeth were relatively fine and uniformly distributed. Numerous reddish chromatophores were observed around the cephalothorax and basal region of the rostrum, although white chromatophores on the dorsal surface were less abundant than in the Na Dun specimen. The rostral formula was 7–11/2–3 teeth. Rostrum length was 5–7 mm (5.99 ± 0.50 mm) as shown in Table 4.

### 3.3. PCR Amplification and DNA Barcodes Identification

**Figure 4:**
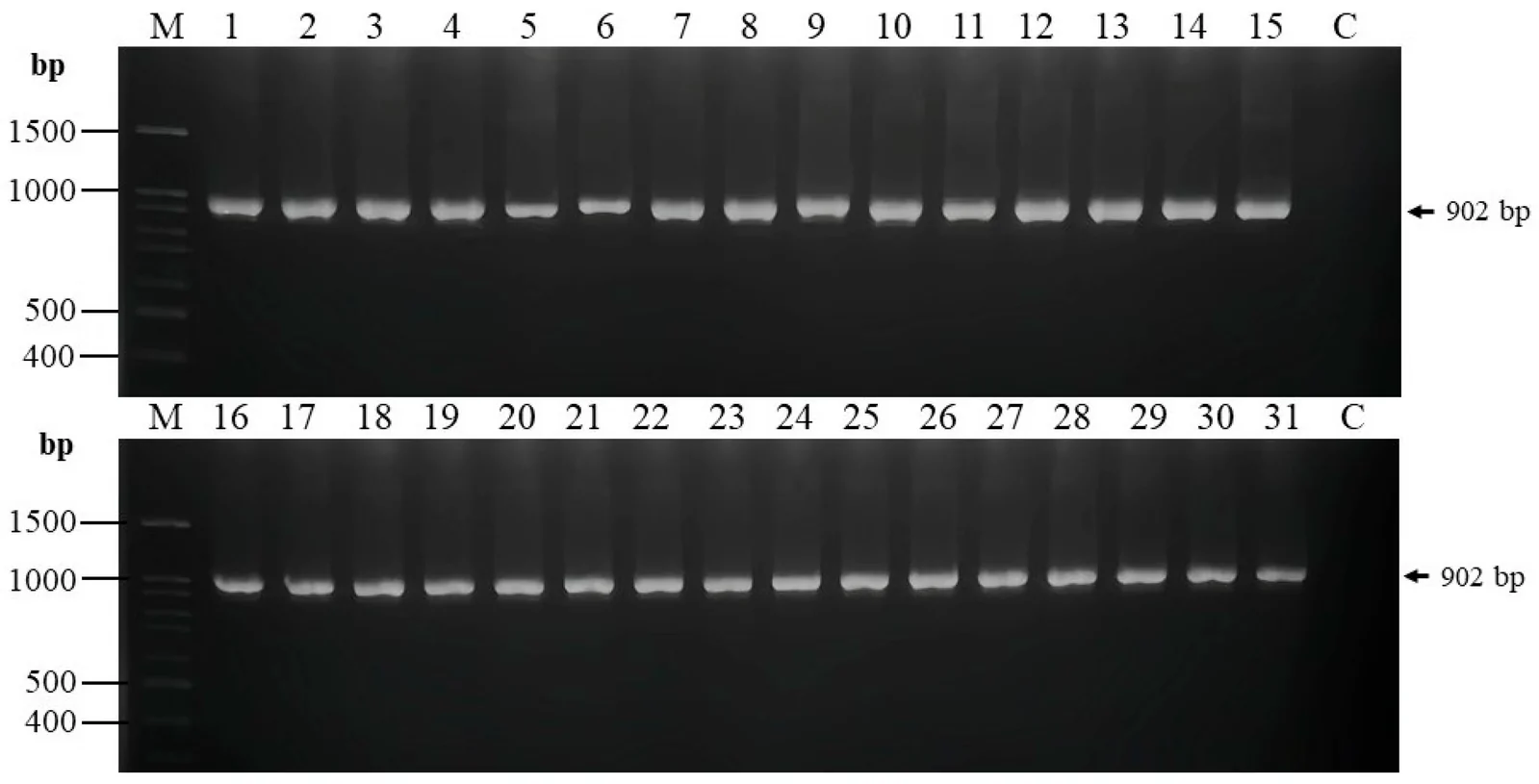
PCR amplification of the 18S rRNA region from freshwater shrimp genomic DNA using the 18S rRNA (F/R) primer pair. Lane M, 100 bp DNA ladder; Lanes 1–5, 17–19, and 24–25, specimens from saline habitats in Wapipathum District; Lanes 6–10, 16, and 26, specimens from saline habitats in Na Dun District; Lanes 11–15, 20–23, and 27–31, specimens from non-saline habitats in Mukdahan Province; Lane C, negative control. M. lanchesteri (Lanes 1–10), M. niphanae (Lanes 11–22), and Caridina sp. (Lanes 23–31) were analyzed. All specimens produced a single amplicon of approximately 902 bp.

**Figure 5:**
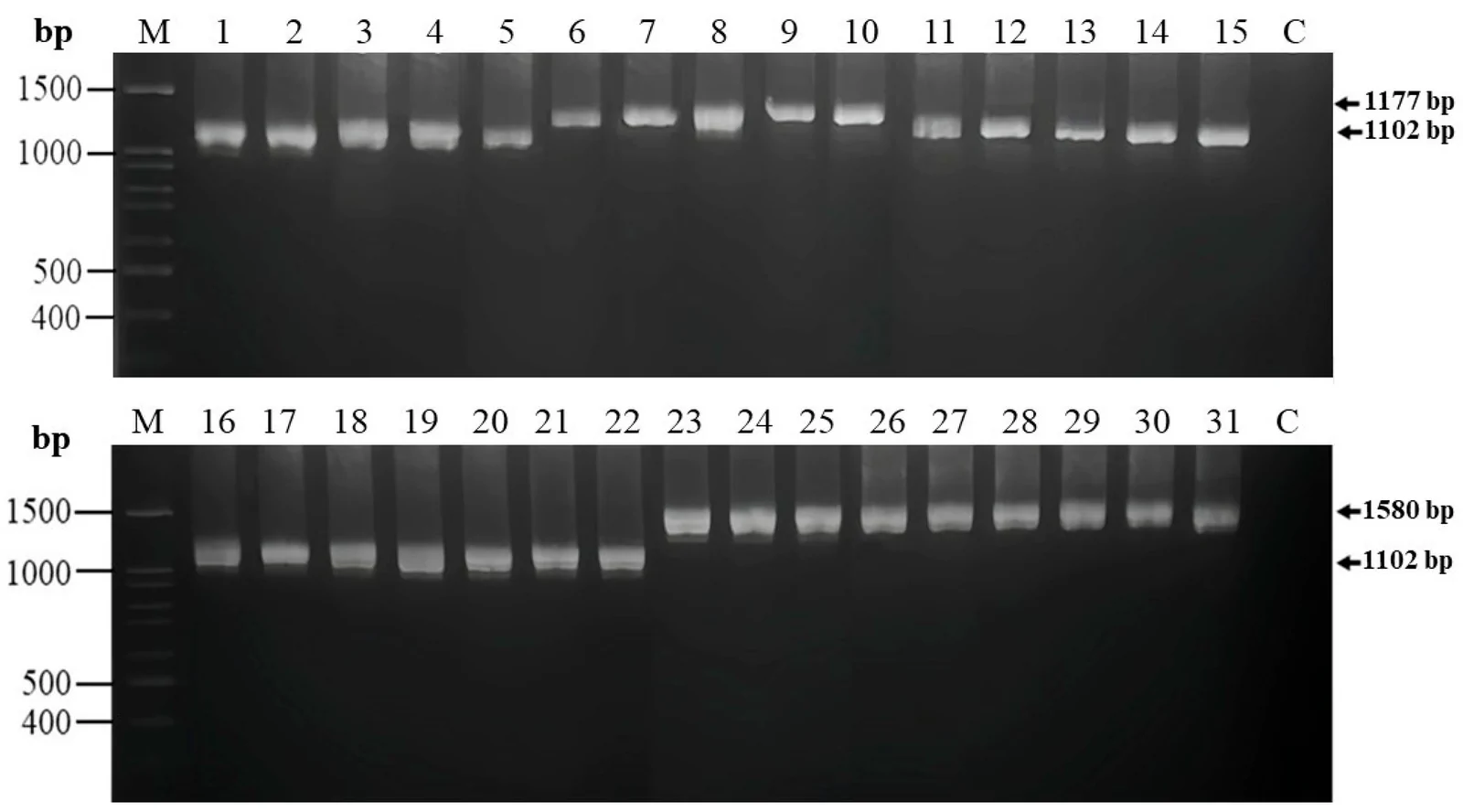
PCR amplification of the ITS region from freshwater shrimp genomic DNA using the ITS (F/R) primer pair. Lane M, 100 bp DNA ladder; Lanes 1–5, 17–19, and 24–25, specimens from saline habitats in Wapipathum District; Lanes 6–10, 16, and 26, specimens from saline habitats in Na Dun District; Lanes 11–15, 20–23, and 27–31, specimens from a non-saline habitat in Mukdahan Province; Lane C, negative control. M. lanchesteri (Lanes 1–10) produced approximately 1102-bp (Lanes 1–5) or 1177-bp (Lanes 6–10) fragments, M. niphanae (Lanes 11–22) approximately 1102-bp fragments, and Caridina sp. (Lanes 23–31) approximately 1580-bp fragments.

The *16S rRNA*, *18S rRNA*, *COI*, and ITS barcode regions were successfully amplified from the freshwater shrimp specimens, with clear PCR products obtained for the analyzed loci. The *18S rRNA* amplification produced a single, well-defined fragment of approximately 902 bp in all 31 specimens (Figure 4). These specimens comprised *M. lanchesteri* (lanes 1–10), *M. niphanae* (lanes 11–22), and *Caridina* sp. (lanes 23–31), collected from saline and non-saline habitats. No amplification was observed in the negative control (lane C), indicating that no detectable PCR contamination occurred. The ITS region also produced distinct amplification patterns among the shrimp taxa (Figure 5). *M. lanchesteri* showed two fragment sizes, with specimens in lanes 1–5 producing approximately 1102 bp amplicons and those in lanes 6–10 producing approximately 1177 bp amplicons. The two ITS fragment-length variants observed in *M. lanchesteri* may reflect either population-level polymorphism or intraindividual variation arising from paralogous ITS copies; however, the present data do not allow these possibilities to be distinguished. *M. niphanae* (lanes 11–22) consistently produced approximately 1102 bp amplicons, whereas *Caridina* sp. (lanes 23–31) produced the largest amplicons, approximately 1580 bp. Thus, the ITS region exhibited clear fragment-length variation associated with the shrimp groups, whereas the 18S rRNA region showed a relatively uniform amplicon size across all specimens. This length polymorphism could reflect either fixed divergence between geographically distinct populations or the presence of intra-individual paralogous ITS copies arising from incomplete concerted evolution within the multicopy rDNA array; the present single-amplicon sequencing approach cannot distinguish between these alternatives.

BLASTn analysis of the obtained barcode sequences revealed high sequence similarity for most *16S rRNA*, *18S rRNA*, and *COI* sequences compared with the closest sequences available in the NCBI GenBank database (Table 5). The *16S rRNA* sequences showed 97–99% identity with *Macrobrachium* and *Caridina* reference sequences, with 98–100% query coverage. The *18S rRNA* sequences showed 97–99% identity and 99–100% query coverage with *Macrobrachium* and *Caridina* sequences. The *COI* sequences generally showed 97–100% identity and 96–100% query coverage; however, the *Caridina sp.* MD sequence showed a lower identity of 86% (497/582 bp) despite a high query coverage of 98%. Notably, the *COI* sequence of *Caridina* sp. M/WP did not yield a significant BLASTn match in the NCBI GenBank database despite successful amplification and sequencing. This may reflect limited reference sequence coverage for *Caridina* and related taxa and/or high sequence polymorphism in the *COI* region. Therefore, species-level identification of this specimen was not assigned based on the *COI* BLASTn result.

In contrast, the ITS sequences did not yield significant or taxonomically informative BLASTn matches in the NCBI GenBank database (Table 5). Therefore, ITS sequence similarity was not used for species identification. Instead, the ITS region was evaluated as a complementary marker based on its sequence variation and fragment-length polymorphism for phylogenetic lineage delineation in combination with the other barcode loci. Overall, the *16S rRNA*, *18S rRNA*, and *COI* sequences provided informative similarity for comparison with reference sequences, whereas ITS contributed complementary variation for resolving phylogenetic relationships among the examined shrimp groups.

### 3.4. Multilocus Phylogenetic Analysis

**Figure 6:**
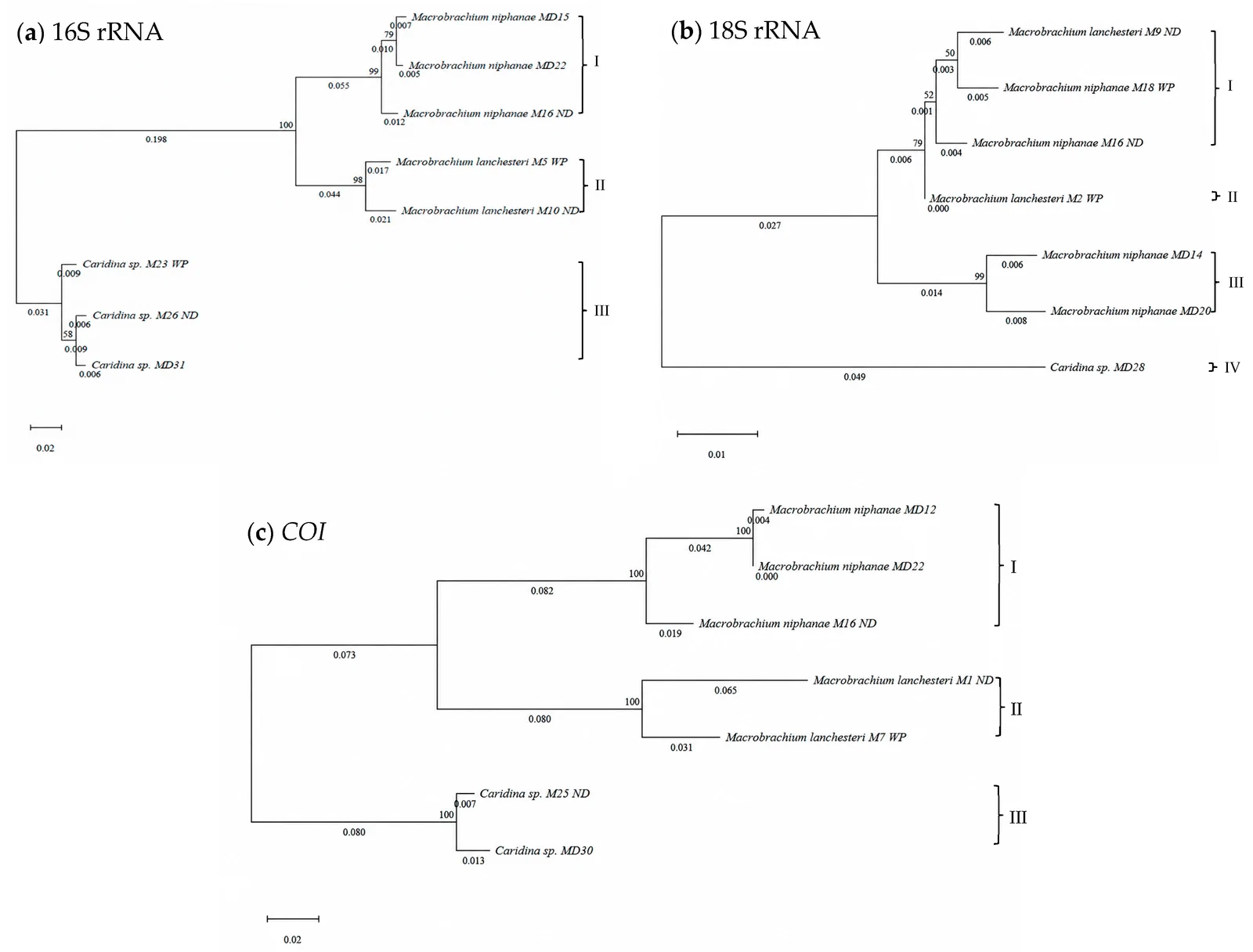

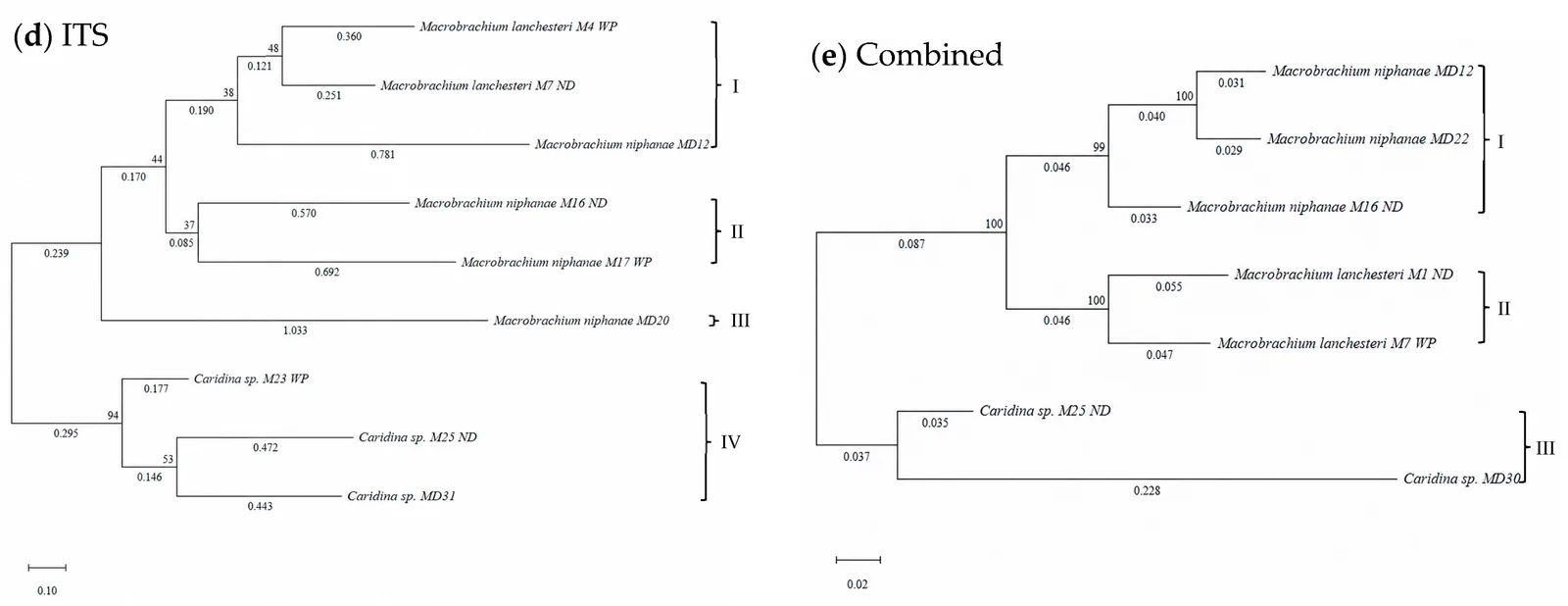
Maximum Likelihood (ML) phylogenetic relationships among 31 shrimp samples inferred from DNA barcode sequences. Phylogenetic trees were reconstructed independently from (**a**) *16S rRNA*, (**b**) *18S rRNA*, (**c**) *COI*, and (**d**) ITS sequences, and from (**e**) a concatenated multilocus dataset comprising all four barcode regions. Numbers at internal nodes represent bootstrap support values (%) based on 1000 replicates. Numbers associated with branches represent branch lengths expressed as nucleotide substitutions per site. The scale bars indicate the corresponding number of substitutions per site.

Phylogenetic relationships among the selected 31 shrimp samples, based on number of species and each species was pickup 3 replicate per one DNA marker for analyses, were reconstructed using ML analyses of the *16S rRNA*, *18S rRNA*, *COI*, and ITS loci, as well as a concatenated dataset comprising all four barcode regions (Figure 6). The ITS dataset was included as a complementary phylogenetic marker to assess sequence variation and support lineage delineation rather than for BLAST-based species identification (Figure 6).

The *16S rRNA* phylogeny resolved three principal groups (Groups I–III) (Figure 6a). Group I comprised the *Macrobrachium niphanae* samples, which formed a closely related lineage, with the terminal relationship between *M. niphanae* MD15 and MD22 receiving strong bootstrap support (BS = 79–100%). Group II consisted of *M. lanchesteri* M5 WP and M10 ND, which formed a distinct and strongly supported lineage (BS = 98%). Group III comprised the three *Caridina* sp. samples (M23 WP, M26 ND, and MD31), which were separated from the *Macrobrachium* lineages by a comparatively long branch. The major separation between the *Macrobrachium* and *Caridina* lineages received maximal bootstrap support (BS = 100%), indicating strong support for their divergence. Branch lengths within the *Macrobrachium* clades were relatively short, whereas the branch separating *Caridina* from *Macrobrachium* was substantially longer (approximately 0.198 substitutions per site), indicating greater sequence divergence between the two genera.

The *18S rRNA* phylogeny showed a somewhat different topology and lower overall resolution than the 16S rRNA tree (Figure 6b). The *Macrobrachium* samples were distributed among several closely related lineages, with bootstrap support ranging from low to high values. The upper portion of the tree included *M. lanchesteri* M9 ND together with *M. niphanae* M18 WP and M16 ND, whereas *M. lanchesteri* M2 WP was positioned within the same broader *Macrobrachium* assemblage. *M. niphanae* MD14 and MD20 formed a separate, strongly supported subclade (BS = 99%). In contrast, *Caridina* sp. MD28 was placed as a distinct basal lineage relative to the *Macrobrachium* samples, with a relatively long branch. These results indicate that 18S rRNA successfully separated the *Caridina* lineage from *Macrobrachium* but provided less consistent discrimination among the *Macrobrachium* samples than the mitochondrial markers.

The *COI* phylogeny produced three clearly differentiated principal groups (Figure 6c). Group I comprised *M. niphanae* MD12, MD22, and M16 ND, with MD12 and MD22 forming a strongly supported sister pair (BS = 100%). Group II consisted of *M. lanchesteri* M1 ND and M7 WP, which formed a distinct monophyletic lineage with maximal bootstrap support (BS = 100%). Group III comprised *Caridina* sp. M25 ND and MD30, which were clearly separated from the *Macrobrachium* lineages. The major *Macrobrachium* groupings were supported by high bootstrap values (BS = 99–100%), demonstrating that COI provided strong resolution of the major taxonomic lineages examined. The relatively short within-group branches further indicated comparatively limited sequence divergence among samples belonging to the same inferred lineage.

The ITS phylogeny exhibited greater variation in branch lengths and comparatively weaker nodal support than the other loci (Figure 6d). Four principal groupings were apparent. The upper lineage contained *M. lanchesteri* M4 WP and M7 ND together with *M. niphanae* MD12, whereas *M. niphanae* M16 ND and M17 WP formed another lineage. *M. niphanae* MD20 was positioned separately, while the three *Caridina* sp. samples formed a distinct lineage at the base of the tree. Bootstrap support for several internal nodes was relatively low (BS = 37–53%), although the *Caridina* lineage was supported by a higher bootstrap value (BS = 94). Clades with bootstrap support below 60% were considered to have limited reliability and were therefore interpreted cautiously. Branch lengths were considerably larger than those observed in the 16S rRNA, 18S rRNA, and *COI* trees, indicating greater sequence divergence in the ITS dataset. However, the relatively low bootstrap support at several internal nodes indicates that the increased sequence divergence did not necessarily translate into stronger phylogenetic resolution.

The concatenated multilocus dataset, combining *16S rRNA*, *18S rRNA*, *COI*, and ITS sequences, produced the most clearly structured overall phylogeny (Figure 6e). Three principal groups were resolved: Group I comprised the *M. niphanae* samples, Group II comprised *M. lanchesteri*, and Group III comprised *Caridina* sp. The *M. niphanae* samples formed a strongly supported lineage, with MD12 and MD22 recovered as a sister pair, while the *M. lanchesteri* samples formed a separate lineage with maximal bootstrap support (BS = 100%). The *Caridina* sp. samples were positioned as a distinct lineage separated from *Macrobrachium* by a comparatively long branch. The major nodes separating the principal lineages received strong support (BS = 99–100%), indicating that the combined dataset provided greater stability and clearer delineation of the principal evolutionary lineages than most individual loci.

The relationship between phylogenetic clustering and habitat type was also examined based on the sampling origins of the specimens. Samples collected from saline habitats in Wapipathum (WP) and Na Dun (ND) districts and those from the non-saline habitat in Mukdahan province (MD) were not consistently separated into distinct phylogenetic lineages. Instead, several phylogenetic groups contained samples originating from different habitat types. For example, in the *16S rRNA* and *COI* trees, *M. niphanae* samples from the non-saline habitat (MD) clustered closely with samples from the saline habitat (ND), while *Caridina* sp. samples from saline (WP and ND) and non-saline (MD) habitats were recovered within the same major lineage. A similar pattern was observed in the concatenated multilocus tree, in which *M. niphanae* MD12 and MD22 from the non-saline habitat clustered with M16 ND from a saline habitat, and *Caridina* sp. M25 ND from a saline habitat clustered with MD30 from a non-saline habitat. These patterns indicate that habitat salinity was not the primary factor determining the major phylogenetic structure of the sampled populations. Nevertheless, the occurrence of closely related samples across contrasting salinity regimes may reflect ecological tolerance, dispersal, historical connectivity, or gene flow between habitats, whereas the distinct clustering of some samples from the same habitat may indicate localized genetic differentiation. Because habitat type was not uniformly concordant with phylogenetic relationships, the present data support an association between genetic structure and taxonomic lineage rather than a direct habitat-driven pattern. Further population-level analyses incorporating larger sample sizes and explicit measures of salinity and genetic differentiation would be required to determine whether environmental salinity contributes to population structuring.

Overall, the multilocus analyses demonstrated that the four barcode regions differed substantially in their ability to resolve relationships among the freshwater shrimp samples. The *16S rRNA* and *COI* datasets provided relatively clear separation of the major *Macrobrachium* and *Caridina* lineages, with particularly strong bootstrap support for the principal nodes. The *18S rRNA* dataset successfully distinguished the *Caridina* lineage but showed greater topological complexity among *Macrobrachium* samples. The ITS dataset exhibited the greatest branch-length variation but relatively weak support for several internal relationships, indicating that greater sequence divergence was not necessarily accompanied by increased phylogenetic confidence. In contrast, the concatenated *16S rRNA* + *18S rRNA* + *COI* + ITS dataset consistently recovered the three principal taxonomic lineages with high bootstrap support, providing the most stable overall framework for assessing phylogenetic relationships among the examined shrimp samples. These findings support the use of ITS as a complementary locus for lineage delineation rather than as a standalone marker for BLAST-based species identification.

### 3.5. Sequence Divergence and Nucleotide Substitution

Pairwise genetic distances estimated using the Kimura two-parameter (K2P) model revealed substantial differences in evolutionary divergence among the four DNA barcode loci (Table 6). The nuclear ITS region exhibited the highest level of sequence variation, with mean intra- and inter-group genetic distances of 1.027 and 1.659, respectively, indicating extensive sequence polymorphism and strong genetic differentiation among shrimp lineages. In contrast, the nuclear *18S rRNA* gene showed the lowest levels of divergence (mean intra-group = 0.041; mean inter-group = 0.043), reflecting its highly conserved evolutionary nature. The mitochondrial *COI* and *16S rRNA* markers displayed intermediate levels of sequence divergence, with *COI* providing moderate variation (mean intra-group = 0.159; mean inter-group = 0.177) and *16S rRNA* exhibiting relatively low within-group divergence but markedly greater between-group divergence (mean intra-group = 0.045; mean inter-group = 0.217). These results demonstrate that each barcode locus contributes different levels of genetic information, with ITS providing the highest discriminatory power, *COI* and *16S rRNA* effectively resolving species-level relationships, and *18S rRNA* serving as a conserved marker for higher-level phylogenetic inference.

Nucleotide substitution probabilities estimated using the Tamura–Nei model are summarized in Table 7. Across all four barcode loci, transition substitutions occurred more frequently than transversions, consistent with substitution patterns commonly observed in mitochondrial and nuclear genomes. The *COI* locus exhibited the highest substitution probability for the C→T transition (20.97%), whereas the *16S rRNA* gene showed the greatest substitution frequency for the G→A transition (21.17%). In the *18S rRNA* locus, substitution probabilities were comparatively uniform, reflecting the conserved nature of this ribosomal gene. The ITS region displayed relatively high transition frequencies, particularly C→T (13.21%) and A→G (13.42%), together with greater overall substitution variability than the other loci. These substitution patterns are consistent with the observed levels of sequence divergence and further demonstrate the contrasting evolutionary rates of mitochondrial and nuclear DNA barcode markers.

ITS produced the greatest absolute difference between mean within- and between-group distances (0.632), followed by *16S rRNA* (0.172), *COI* (0.018) and *18S rRNA* (0.002). ITS nevertheless retained a partial overlap between 1.441 and 1.665, indicating strong but incomplete lineage separation. Despite the overall high divergence of ITS, this partial overlap indicates that ITS alone cannot provide definitive species delimitation and should therefore be interpreted as a complementary marker for lineage delineation. The broad *16S rRNA* inter-group range of 0.012–0.277 indicated heterogeneous divergence, including some between-group comparisons that were less divergent than within-group comparisons. *COI* and *18S rRNA* showed extensive overlap and consequently provided limited barcode-gap resolution.

## 4. Discussion

Salinity may reflect gene diversity through several non-exclusive processes. For example: Frist, species differ in osmoregulatory capacity, so elevated salinity and conductivity may selectively exclude sensitive lineages while allowing ecological generalists to persist. Second, energetic investment in ion regulation may reduce growth, reproduction or larval recruitment. Third, saline-soil habitats were more physiochemically variable, particularly in EC and pH, potentially producing less stable environmental conditions. The differences of four gene maker could reflect shared ancestral variation, historical watershed connectivity, occasional dispersal or anthropogenic movement. Historical changes in hydrological connectivity can produce genetic patterns that persist long after populations become geographically isolated, as demonstrated in phylogeographic studies of *Macrobrachium* species [35]. Therefore, the observed clustering should not be described as direct evidence of contemporary salinity-driven isolation.

Although these differences in rostral morphology were consistently observed among populations, the diagnostic characters overlapped considerably, making reliable species identification based solely on rostral morphology difficult. Variation in rostral shape and dentition has been reported in several *Caridina* species and may reflect geographic variation, environmental conditions, developmental stage, or phenotypic plasticity rather than species-level divergence [36]. Previous studies of freshwater *Caridina* have demonstrated that robust species delimitation benefits from concordance among mitochondrial DNA, independent nuclear loci, multiple species-delimitation methods, geographic distributions and morphological evidence [37,38,39].

Seasonal hydrology must be considered when interpreting the difference between saline and non-saline sites. Northeastern Thailand experiences pronounced wet and dry seasons that affect precipitation, stream discharge, water depth, groundwater–surface-water exchange, and dissolved-ion concentrations. Conductivity in regional rivers generally increases as water levels decline and decreases when higher discharge dilutes dissolved ions [36]. Because northeastern Thailand experiences pronounced seasonal hydrological fluctuation, with conductivity and salinity typically peaking during low-flow dry-season conditions and declining as wet-season discharge dilutes dissolved ions [40]. The environmental contrasts observed between saline and non-saline sites in this study represent a single seasonal snapshot rather than a stable, year-round condition. Wet-season sampling could reveal a different salinity/conductivity profile at the same sites, and increased hydrological connectivity during flooding may facilitate gene flow between populations that appear isolated under dry-season conditions [41]. This temporal limitation further reinforces our conclusion that the observed genetic clustering should not be interpreted as direct evidence of contemporary, salinity-driven population isolation, and highlights the need for multi-seasonal sampling in future studies to disentangle short-term environmental variation from more stable historical or evolutionary signals [42].

Therefore, rostral morphology alone was insufficient for species delimitation in the present study, emphasizing the necessity of integrating molecular evidence, particularly *COI* DNA barcoding, with traditional morphological identification to resolve cryptic diversity. The present study demonstrates that different DNA barcode markers provide complementary levels of taxonomic resolution. *COI*, the internationally accepted standard barcode for animals [43], reliably identified species boundaries but showed limited ability to resolve fine-scale geographic divergence. In contrast, *16S rRNA* detected additional phylogeographic structure, whereas the nuclear markers, particularly ITS, exhibited the highest discriminatory power for distinguishing closely related populations. The ability of ITS to recover six distinct lineages suggests that nuclear ribosomal markers may retain informative variation overlooked by mitochondrial loci, particularly among recently diverged populations or incipient cryptic species.

Importantly, although *COI* and *16S rRNA* are widely employed in crustacean taxonomy [44,45,46,47,48], the application of *18S rRNA* and ITS for freshwater shrimp identification in Thailand has been very limited.

The contrasting amplification patterns observed for the *18S rRNA* and ITS loci provide insight into the different evolutionary constraints acting on nuclear ribosomal DNA. The highly uniform amplification profile of the *18S rRNA* gene across all freshwater shrimp specimens is consistent with its conserved structural and functional role in ribosome biogenesis, where strong purifying selection restricts both nucleotide substitutions and insertion–deletion events [49]. In contrast, the ITS region exhibited marked fragment-length polymorphism among the examined species, reflecting the relaxed selective constraints acting on this non-coding spacer. Variation in ITS length is commonly generated through insertion–deletion mutations and incomplete concerted evolution within multicopy rDNA arrays, resulting in structural polymorphism among closely related taxa [48,49,50]. The occurrence of two ITS length variants in *M. lanchesteri*, together with the distinct fragment profile observed in *Caridina* sp., suggests that ITS evolution has proceeded more rapidly than that of the conserved ribosomal genes and may retain signatures of recent lineage divergence that are not detectable using coding loci alone.

These differences in molecular evolution were reflected in the phylogenetic reconstructions. The mitochondrial *COI* marker provided the clearest species discrimination and strongest nodal support, whereas *16S rRNA* generated a comparable topology but with lower sequence divergence. The higher resolving power of *COI* is expected because mitochondrial protein-coding genes generally evolve faster than ribosomal genes and are inherited as a non-recombining maternal genome, facilitating more rapid lineage sorting following species divergence [51,52]. Conversely, the highly conserved *18S rRNA* gene preserved deep phylogenetic relationships but contained relatively few informative substitutions, whereas the structurally variable ITS region recovered additional lineages that were less apparent from mitochondrial markers. Similar observations have been reported in *M. lanchesteri*, where ITS2 resolved geographically structured haplotypes that could not be distinguished using morphology or mitochondrial DNA alone [53]. Together, these findings demonstrate that mitochondrial and nuclear loci capture complementary components of evolutionary history because they evolve under different mutation rates, inheritance patterns, and selective pressures.

The sequence divergence and nucleotide substitution analyses further support these contrasting evolutionary patterns. The greater genetic divergence and substitutional variability observed in the ITS region indicate rapid molecular evolution and the accumulation of numerous phylogenetically informative characters, whereas the low divergence of 18S rRNA reflects strong functional constraint. The predominance of transition substitutions over transversions across all four loci is consistent with the general expectations of nucleotide evolution under the Tamura–Nei model, in which chemically similar substitutions occur more frequently than transversions because they are less likely to disrupt DNA structure and function [54,55]. Importantly, integrating mitochondrial (*COI* and *16S rRNA*) and nuclear (*18S rRNA* and ITS) markers substantially increased phylogenetic resolution and bootstrap support compared with individual loci, demonstrating that multilocus DNA barcoding reduces locus-specific bias and provides a more reliable estimate of species relationships [56]. This integrative approach therefore offers a robust molecular framework for species identification, phylogenetic reconstruction, and future biodiversity and conservation studies of freshwater shrimps. However, the gene tree discordance observed among the four barcode loci particularly the contrasting topologies recovered by the mitochondrial versus nuclear markers could arise from either incomplete lineage sorting (ILS) following rapid diversification, or historical hybridization/introgression between lineages, or a combination of both processes. Because both processes can produce statistically indistinguishable patterns of gene tree conflict when only a small number of loci are available [57].

To our knowledge, this is the first study to directly compare mitochondrial and nuclear DNA barcodes for freshwater prawns inhabiting saline and non-saline ecosystems in northeastern Thailand. The superior resolution obtained with ITS highlights its potential as a complementary marker for integrative taxonomy, phylogeography, and the detection of cryptic diversity in *Macrobrachium* and *Caridina*. Consequently, combining mitochondrial and nuclear markers is recommended for future biodiversity assessments and species delimitation studies rather than relying exclusively on *COI* DNA barcoding. Validation of the ITS lineages was constrained by the scarcity of taxonomically verified ITS sequences for Thai *Caridina* and *Macrobrachium* in public databases. Future work should generate voucher-linked ITS1–5.8S–ITS2 reference sequences from morphologically verified specimens, including topotypical material where possible. Sequences should be deposited with specimen photographs, collection coordinates, habitat data and museum voucher numbers. Because ITS is multicopy and may exhibit intra-individual variants, cloning or high-throughput amplicon sequencing should be used to detect paralogues and divergent ribosomal copies. Expanding this reference library would improve species authentication and determine whether the six candidate ITS lineages represent reproductively independent species or geographically structured populations.

## 5. Conclusions

The four DNA barcode markers displayed complementary advantages in freshwater shrimp identification and phylogenetic inference. The mitochondrial *COI* gene was the most reliable marker for routine species identification due to its consistent application, wide worldwide reference databases (GenBank and BOLD) and effective species-level discrimination. In contrast, *16S rRNA* was better at delineating intraspecific and regional variation than COI, and was thus particularly valuable for population-level studies. The nuclear *18S rRNA* gene is relatively preserved, yet it resolved key evolutionary lineages and provided an independent source of phylogenetic data complementary to mitochondrial markers. Among the four markers, the nuclear ITS region exhibited the greatest sequence variation and was useful for identifying candidate genetic lineages and supporting lineage delineation. However, ITS alone cannot provide definitive species delimitation because of partial overlap in genetic distances, limited bootstrap support for some internal clades, and the potential for intraindividual paralogous copies. Therefore, ITS-based lineage interpretation should be integrated with mitochondrial markers, particularly *COI*, and morphological evidence for robust species-level inference. *COI* is suitable for routine species identification; ITS is useful for discovering candidate genetic lineages; ITS alone cannot accomplish species delimitation, and multisource evidence needs to be integrated, thereby providing new insights into freshwater shrimp biodiversity and phylogenetic relationships across saline and non-saline habitats and contributes to a more comprehensive understanding of freshwater shrimp biodiversity and conservation in the Mekong region.

## Figures and Tables

**Table 1:** Sampling localities and distribution of 225 freshwater shrimp specimens from 10 sites in Mahasarakham (M) and 5 sites in Mukdahan (MD) Provinces, Thailand.

Station No.	Area	Province, Abbreviations	Locality Description	Water Quality				GPS Coordinates
				Temperature (°C)	pH	Salinity (ppt)	EC (µS/cm)	
1	Saline soil	Mahasarakham, M	Na Dun1	27	6.8	0.15	524	15°41′51.01″ N, 103°13′53.30″ E
2		Mahasarakham, M	Na Dun2	24	8.9	0.125	581	15°41′51.02″ N, 103°13′53.31″ E
3		Mahasarakham, M	Na Dun3	28	6.9	0.15	578	15°41′52.40” N, 103°13′50.19″ E
4		Mahasarakham, M	Na Dun4	27	6.6	0.15	631	15°41′16.41″ N, 103°14′30.56″ E
5		Mahasarakham, M	Na Dun5	30.5	7.1	0.1	402	15°41′18.48″ N, 103°15′01.03″ E
6		Mahasarakham, M	Wapipatum1	32.6	5.5	0.5	2050	15°49′11.74″ N, 103°19′30.65″ E
7		Mahasarakham, M	Wapipatum2	30.5	6.7	0.3	879	15°49′43.81″ N, 103°21′15.56″ E
8		Mahasarakham, M	Wapipatum 3	30.5	6.6	0.25	751	15°49′30.86″ N, 103°20′48.74″ E
9		Mahasarakham, M	Wapipatum4	31.5	5.0	0.40	592	15°49′26.62″ N, 103°20′47.99″ E
10		Mahasarakham, M	Wapipatum5	26.3	7.7	0.55	921	15°48′32.00″ N, 103°20′27.00″ E
11	Non-saline soil	Mukdahan, MD	Mekong River Basin1	29.1	7.5	0.05	280	16°28′54.80″ N, 104°42′45.70″ E
12		Mukdahan, MD	Mekong River Basin2	24.5	8.5	0.05	305	16°31′02.00″ N, 104°44′44.00″ E
13		Mukdahan, MD	Mekong River Basin3	29.0	7.5	0.00	252	16°30′33.79″ N, 104°43′22.24″ E
14		Mukdahan, MD	Mekong River Basin4	25.0	7.6	0.05	298	16°28′55.44″ N, 104°42′49.39″ E
15		Mukdahan, MD	Mekong River Basin5	30.0	8.8	0.01	245	16°30′30.21″ N, 104°43′10.65″ E

**Note:** Numbers 1–5 indicate the individual sampling sites within each study area. Na Dun1–Na Dun5, Wapi Pathum1–Wapi Pathum5, and Mekong River Basin1–Mekong River Basin5.

**Table 2:** Primer sequences and optimized annealing temperatures used for amplification of the *COI*, 16S rRNA, 18S rRNA, and ITS DNA barcode regions analyzed in this study.

DNA Barcode	Primer Sequence (5′–3′)	Annealing (°C)	Reference
*COI*	Forward: LCO1490, 5′-GGTCAACAAATCATAAAGATATTGG-3′ Reverse: MacroNancy, 5′-GCGGGTAGRATAARATRTATACTTC-3′	52	[26]
*16S rRNA*	Forward: 16Sa-L, 5′-CGCCTGTTTATCAAAAACAT-3′ Reverse: 16Sbr-H2, 5′-CTCCGGTTTGAACTCAGATCA-3′	53	[27]
*18S rRNA*	Forward: 18S-ai, 5′-CCTGAGAAACGGCTACCACATC-3′ Reverse: 18S-bi, 5′-GAGTCTCGTTCGTTATCGGA-3′	55	[28]
ITS	Forward: ITS5, 5′-GGAAGTAAAAGTCGTAACAAGG-3′ Reverse: ITS4, 5′-TCCTCCGCTTATTGATATGC-3′	55	[29]

Note: PCR amplification was performed using the same reaction mixture and cycling profile for all loci, with annealing temperatures optimized according to each primer pair.

**Table 3:** Water quality in Mahasarakham and Mukdahan provinces, Thailand.

Parameter	Maha Sarakham: Saline-Soil Sites (Mean ± SD)	Mukdahan: Non-Saline Sites (Mean ± SD)	Ecological Interpretation
Temperature (°C)	28.79 ± 2.72 ^a^	27.52 ± 2.56 ^b^	Maha Sarakham had a wider thermal range and a slightly higher mean temperature
pH	6.78 ± 1.07 ^b^	7.98 ± 0.62 ^a^	Maha Sarakham ranged from acidic to alkaline; Mukdahan was consistently neutral–alkaline
Salinity (ppt)	0.268 ± 0.16 ^a^	0.032 ± 0.02 ^b^	Mean salinity was approximately eight times higher in Maha Sarakham
EC (µS/cm)	791 ± 47.00 ^a^	276 ± 26.80 ^b^	Maha Sarakham contained substantially more dissolved ions and showed greater spatial heterogeneity
Water appearance	Clear to turbid; green, brown, black, or tea-colored	Mostly clear; occasionally green or tea-colored	Differences may reflect algae, suspended matter, dissolved organic matter, and local habitat conditions
Visible habitat features	Algae, periphyton, duckweed, grass, and sunlight exposure	Algae, aquatic plants, grass, and sunlight exposure	Both areas provided vegetation and detrital microhabitats, but habitat structure varied among localities

Values with different letters are significantly different at *p* < 0.05.

**Table 4:** Quantitative rostral metrics (carapace length, rostrum length, and rostrum-to-carapace length ratio) of three freshwater shrimp species across sampling areas in Mahasarakham (saline) and Mukdahan (non-saline) provinces, Thailand (mean ± SD, *n* = 3 per group). Values with different superscript letters within the same species are significantly different *p* < 0.05.

Species	Site	CL (mm)	RL (mm)	RL/CL Ratio
*Caridina* sp.	Mukdahan (non-saline)	6.35 ± 0.97	5.76 ± 0.79	0.93 ± 0.27 ᵃ
	Na Dun (saline)	4.97 ± 0.48	4.53 ± 0.25	0.91 ± 0.04 ᵃ
	Wapipathum (saline)	4.45 ± 0.36	3.59 ± 0.80	0.80 ± 0.13 ᵃ
*M. lanchesteri*	Mukdahan (non-saline)	8.57 ± 0.10	8.38 ± 0.03	0.98 ± 0.01 ᵇ
	Na Dun (saline)	5.08 ± 0.10	6.27 ± 0.67	1.23 ± 0.11 ᵃ
	Wapipathum (saline)	3.81 ± 0.12	3.57 ± 0.08	0.94 ± 0.04 ᵇ
*M. niphanae*	Mukdahan (non-saline)	9.47 ± 0.28	5.99 ± 0.50	0.63 ± 0.04 ᵇ
	Na Dun (saline)	11.90 ± 0.68	11.84 ± 1.28	0.99 ± 0.07 ᵃ
	Wapipathum (saline)	11.73 ± 0.40	7.07 ± 0.23	0.60 ± 0.02 ᵇ

**Table 5:** BLASTn sequence similarity analysis of DNA barcode loci and their closest matches retrieved from the NCBI GenBank database.

Sample	Barcode	Accession No.	Closest Match (GenBank)	Match Accession No.	Aligned Length (bp)	Identity	% Match	Coverage (%)	E-Value
1. *Macrobrachium lanchesteri* M/WP	*16S*	PZ428996	*Macrobrachium* sp.	OR578655.1	521	519/521	99	99	0.0000
	*18S*	PZ428978	*Macrobrachium lanchesteri*	KP725754.1	926	924/926	99	100	0.0000
	*COI*	PZ441256	*Macrobrachium* sp.	OR575114.1	604	594/604	98	96	0.0000
	ITS	PZ465419	*-*	-	-	-	-	-	-
2. *Macrobrachium lanchesteri* M/ND	*16S*	PZ428997	*Macrobrachium lanchesteri*	OR578691.1	522	518/522	99	99	0.0000
	*18S*	PZ428979	*Macrobrachium lanchesteri*	KP725754.1	939	933/939	99	99	0.0000
	*COI*	PZ441257	*Macrobrachium lanchesteri*	MW845496.1	623	623/623	100	100	0.0000
	ITS	PZ465420	*-*	-	-	-	-	-	-
3. *Macrobrachium niphanae* MD	*16S*	PZ428998	*Macrobrachium niphanae*	JQ359752.1	521	513/521	98	98	0.0000
	*18S*	PZ428980	*Macrobrachium niphanae*	MF622008.1	926	921/926	99	99	0.0000
	*COI*	PZ441258	*Macrobrachium niphanae*	PV651615.1	531	529/531	99	100	0.0000
	ITS	PZ465421	*-*	-	-	-	-	-	-
4. *Macrobrachium niphanae* M/ND	*16S*	PZ428999	*Macrobrachium niphanae*	JQ359752.1	511	502/511	98	99	0.0000
	*18S*	PZ428981	*Macrobrachium* sp.	MH053370.1	926	924/926	99	99	0.0000
	*COI*	PZ441259	*Macrobrachium niphanae*	MW845633.1	591	580/591	98	99	0.0000
	ITS	PZ465422	*-*	-	-	-	-	-	-
5. *Macrobrachium niphanae* M/WP	*16S*	PZ429000	*Macrobrachium* sp.	OR578690.1	513	502/513	98	99	0.0000
	*18S*	PZ428982	*Macrobrachium* sp.	MH053370.1	949	941/949	99	99	0.0000
	*COI*	PZ441260	*Macrobrachium lanchesteri*	MW845496.1	623	623/623	100	100	0.0000
	ITS	PZ465423	*-*	-	-	-	-	-	-
6. *Macrobrachium niphanae* MD	*16S*	PZ429001	*Macrobrachium niphanae*	JQ359752.1	510	504/510	99	99	0.0000
	*18S*	PZ428983	*Macrobrachium niphanae*	MT248185.1	918	915/918	99	99	0.0000
	*COI*	PZ441261	*Macrobrachium* sp.	MW845561.1	588	584/588	99	100	0.0000
	ITS	PZ465424	*-*	-	-	-	-	-	-
7. *Caridina* sp. M/ND	*16S*	PZ429002	*Caridina nilotica macrophora*	PX833795.1	528	511/528	97	100	0.0000
	*18S*	PZ428984	*Caridina* sp.	MF622000.1	922	919/922	99	99	0.0000
	*COI*	PZ441262	*Caridina* sp.	PV540209.1	546	528/546	97	100	0.0000
	ITS	PZ465425	*-*	-	-	-	-	-	-
8. *Caridina* sp. M/WP	*16S*	PZ429003	*Caridina nilotica macrophora*	PX833795.1	518	509/518	98	98	0.0000
	*18S*	PZ428985	*Caridina serratirostris*	KP725709.1	943	918/943	97	99	0.0000
	*COI*	-	*-*	-	-	-	-	-	-
	ITS	PZ465426	*-*	-	-	-	-	-	-
9. *Caridina* sp. MD	*16S*	PZ429004	*Caridina nilotica macrophora*	PX833795.1	527	518/527	98	100	0.0000
	*18S*	PZ428986	*Caridina* sp.	MF622000.1	926	923/926	99	100	0.0000
	*COI*	PZ441263	Caridina sp.	MK190019.1	582	497/582	86	98	7 × 10^−167^
	ITS	PZ465427	-	-	-	-	-	-	-

Note: BLASTn searches were performed against the NCBI GenBank database. ITS sequences yielded no significant matches and therefore no reliable similarity or taxonomic assignment. Identity is reported as the number of identical nucleotides relative to the aligned length, with percentages in parentheses. An hyphen (-) indicates no significant BLASTn match or no sequence available.

**Table 6:** Estimates of intra- and inter-group evolutionary divergence for four DNA barcode loci (COI, 18S, 16S, and ITS) in shrimps.

Parameter	*COI*	*18S rRNA*	*16S rRNA*	ITS
Range of intra-group genetic distance	0.010–0.250	0.010–0.091	0.020–0.076	0.605–1.665
Range of inter-group genetic distance	0.014–0.244	0.019–0.055	0.012–0.277	1.441–1.890
Mean intra-group genetic distance	0.159	0.041	0.045	1.027
Mean inter-group genetic distance	0.177	0.043	0.217	1.659

Evolutionary divergence estimates were calculated using the Kimura 2-parameter model implemented in MEGA version 11.

**Table 7:** Estimated nucleotide substitution probabilities (%) among four DNA barcode loci (COI, 18S rRNA, 16S rRNA, and ITS) in freshwater shrimps.

From/To	*COI*				*18S rRNA*				*16S rRNA*				ITS			
	A	T	C	G	A	T	C	G	A	T	C	G	A	T	C	G
A	-	6.286	4.826	8.435	-	6.343	6.418	12.845	-	7.712	2.627	16.223	-	6.583	5.574	13.421
T	5.781	-	16.101	3.620	7.507	-	9.352	7.943	6.271	-	5.025	4.806	6.115	-	11.188	6.595
C	5.781	20.972	-	3.620	7.507	9.242	-	7.943	6.271	14.754	-	4.806	6.115	13.213	-	6.595
G	13.467	6.286	4.826	-	12.140	6.343	6.418	-	21.168	7.712	2.627	-	12.443	6.583	5.574	-

Note: Substitution probabilities were estimated under the Tamura-Nei (TN93) model. Transitional substitutions (A↔G and C↔T) are shown in bold, whereas transversional substitutions are presented in regular font. Values represent the relative substitution probabilities (%) estimated from the aligned barcode sequences.

## Data Availability

The data presented in this study are available on request from the corresponding author due to privacy.

## Abbreviations

The following abbreviations are used in this manuscript:

M	Mahasarakham province
MD	Mukdahan province
ND	Nadun district
WP	Wapipathum district
